# Air mass advection and immune dysregulation in schizophrenia: an exploratory pilot study

**DOI:** 10.3389/fpsyt.2026.1950134

**Published:** 2026-10-19

**Authors:** Stefan Modzelewski, Aleksandra Julia Oracz, Maria Suprunowicz, Aleksandra Śladewska, Oliwia Froń, Jakub Wiktor Kosidło, Joanna Matowicka-Karna, Napoleon Waszkiewicz

**Affiliations:** 1 Department of Psychiatry, Medical University of Białystok, Białystok, Poland; 2 Clinical Laboratory Diagnostics Facility, Medical University of Białystok, Białystok, Poland

**Keywords:** air masses, inflammation, psychopathology, schizophrenia, seasonality, weather

## Abstract

**Background:**

Schizophrenia is a severe and clinically heterogeneous mental disorder with an unpredictable course. Growing evidence indicates that immune dysregulation and environmental factors, including weather conditions, may influence symptom exacerbations. Although seasonality has been repeatedly described, the impact of specific meteorological variables - particularly wind and air masses - remains insufficiently explored, and biochemical correlates of weather exposure are rarely examined, despite the fact that wind could inherently convey a complex suite of region-specific meteorological data, thereby offering a more holistic and integrated index of environmental exposure. This exploratory pilot study evaluated two distinct biometeorological approaches - local wind vector decomposition versus macro-scale air mass advection - to examine their associations with psychopathology and cytokine profiles in patients with schizophrenia.

**Methods:**

This cross-sectional observational study included a total cohort of 104 participants, consisting of 19 healthy controls and 85 patients with schizophrenia. Psychopathology was evaluated using the Positive and Negative Syndrome Scale (PANSS). Serum levels of TNF-α, IFN-γ, IL-6, and IL-4 were measured and analyzed for 84 subjects, comprising 19 controls and 65 patients with schizophrenia. Weather exposure was analyzed using two models: (1) a local approach, decomposing wind direction into meridional and zonal vector components using trigonometric transformation (3-day average) and (2) a synoptic approach, calculating 7-day backward air mass trajectories, categorized into Arctic, Polar Continental, Polar Maritime, and Tropical types.

**Results:**

Among patients with schizophrenia, Tropical air masses were associated with lower IFN-γ and TNF-α concentrations than all other air mass types (Kruskal–Wallis p = 0.016 and p = 0.001, respectively; all adjusted *post-hoc* p < 0.05). The association between air mass type and ln(IFN-γ) differed by health status: Tropical air masses were associated with lower ln(IFN-γ) in patients but higher ln(IFN-γ) in healthy controls compared with Polar Maritime and Arctic air masses (both interaction contrasts p < 0.001). Patients had higher IFN-γ than controls under polar maritime and Arctic air masses, whereas no between-group difference was observed under Tropical air masses. Neither air mass type nor continuous wind-vector components were associated with PANSS scores.

**Conclusion:**

Patients with schizophrenia and healthy controls exhibited directionally opposite IFN-γ patterns across air mass types, suggesting health-status-dependent immune responsiveness to synoptic environmental conditions. This pilot study highlights that macro-scale air mass advection, rather than local wind direction components, may represent a more informative environmental proxy for detecting weather-induced immune modulation. Although this study is constrained by a major limitation regarding sample size, these preliminary findings warrant further investigation.

## 1 Introduction

Schizophrenia is one of the most severe mental disorders, frequently associated with deterioration of patient functioning across nearly all domains of life. Despite advances in understanding this condition, accurate prediction of disease exacerbations remains elusive, likely due to the multifactorial nature of schizophrenia and the substantial heterogeneity of its clinical presentation across patients. Current clinical knowledge identifies several important factors associated with relapse or symptom exacerbation in schizophrenia, including medication discontinuation and exposure to psychosocial stressors (1, 2). Nevertheless, the clinical course of the disorder often remains difficult to predict. Therefore, there is a need to search for clinically useful predictors and biomarkers in psychiatry that could help clinicians and healthcare systems identify patterns of exacerbation leading to hospitalization. The lack of such tools contributes to high treatment costs, progressive loss of quality of life with each psychotic episode, and difficulties in selecting optimal treatment strategies (3, 4).

Most previous attempts to identify biomarkers or predictors of schizophrenia course have focused on conventional diagnostic domains, including neuroimaging and peripheral blood markers (5, 6). In this context, several studies have reported alterations in peripheral cytokine levels in patients with schizophrenia (7, 8), potentially reflecting an ongoing neuroinflammatory process (9), Such immune dysregulation may represent one biological factor contributing to the clinical heterogeneity and unpredictability of the disorder (10). A separate and less extensively studied line of evidence concerns the seasonal and cyclical aspects of schizophrenia. This includes observations of non-random birth-month distributions among individuals with schizophrenia, with clustering commonly reported in autumn and winter periods, which are generally characterized by lower ambient temperatures (11). An additional aspect of this complexity is the repeatedly observed seasonality of schizophrenia, where specific weather factors and seasonal patterns have been linked to the course of schizophrenia and other psychiatric disorders. Most studies in this area primarily focus on meteorological factors as risk determinants for aggressive or risky behaviors or attempt to assess overall epidemiological trends for a given disorder, while less frequently examining their influence on the clinical characteristics of the illness itself (12–14).

Furthermore, the mechanisms through which meteorological factors may influence the clinical state of patients remain largely unknown. One conceptual framework proposed to explain the potential impact of weather on psychiatric symptoms is immunoseasonality. According to this hypothesis, weather-related factors may modulate immune system activity, leading to an overall deterioration in patients’ clinical condition (15).

However, despite a large body of observational research investigating associations between weather and mental health, biochemical parameters such as cytokines have rarely been examined in patients exposed to specific meteorological conditions (12), even though such measurements are essential for testing the biological assumptions underlying the immunoseasonal theory. Moreover, relatively few studies have focused on wind (16). Notably, existing studies have most often examined only very specified wind types, such as mountain winds (17). Although seasonality and selected meteorological variables have been investigated in relation to schizophrenia admissions and symptom variability, the potential role of synoptic air mass types in shaping the clinical course or symptom characteristics of schizophrenia remains largely unexplored. Air mass classification may offer a more comprehensive representation of environmental exposure because it integrates several co-occurring meteorological features into a single synoptic category, rather than considering temperature, humidity, pressure, or wind direction separate (18–20).

In this cross-sectional exploratory study, we aimed to evaluate the relationships between meteorological exposure, clinical symptom severity, and cytokine profiles in patients with schizophrenia. Meteorological exposure was assessed using two complementary approaches: a continuous local wind-vector decomposition model, capturing wind inflow direction as meridional and zonal components, and a categorical synoptic air-mass classification model, reflecting the broader origin and characteristic meteorological properties of moving air masses.

## 2 Materials and methods

This cross-sectional observational study enrolled 104 participants: 85 patients with schizophrenia and 19 healthy controls, between January 2025 and June 2026. All research activities - comprising informed consent, blood collection, and clinical evaluation - were performed during a single session in either inpatient or outpatient settings (**Figure 1**). The study adhered to the Declaration of Helsinki and received ethical clearance from the Medical University of Białystok Bioethics Committee (ref. no. APK.002.528.2024). Participants provided written informed consent following a comprehensive briefing on the study’s aims. The present study builds upon and extends our previous investigation (21). Although the two studies partially overlap in their patient samples and use selected cytokine measurements obtained from the same baseline blood samples, their final analytic samples differ. The current study includes additional patients and a healthy control group and broadens the cytokine assessment by incorporating IL-4, which was not reported in the previous study. It also extends the earlier work methodologically by applying wind-vector modeling and trajectory-based synoptic air-mass classification and by examining whether IFN-γ patterns across air mass types differ according to health status. These air-mass-based analyses and patient-control comparisons have not been reported previously.

**Figure 1:**
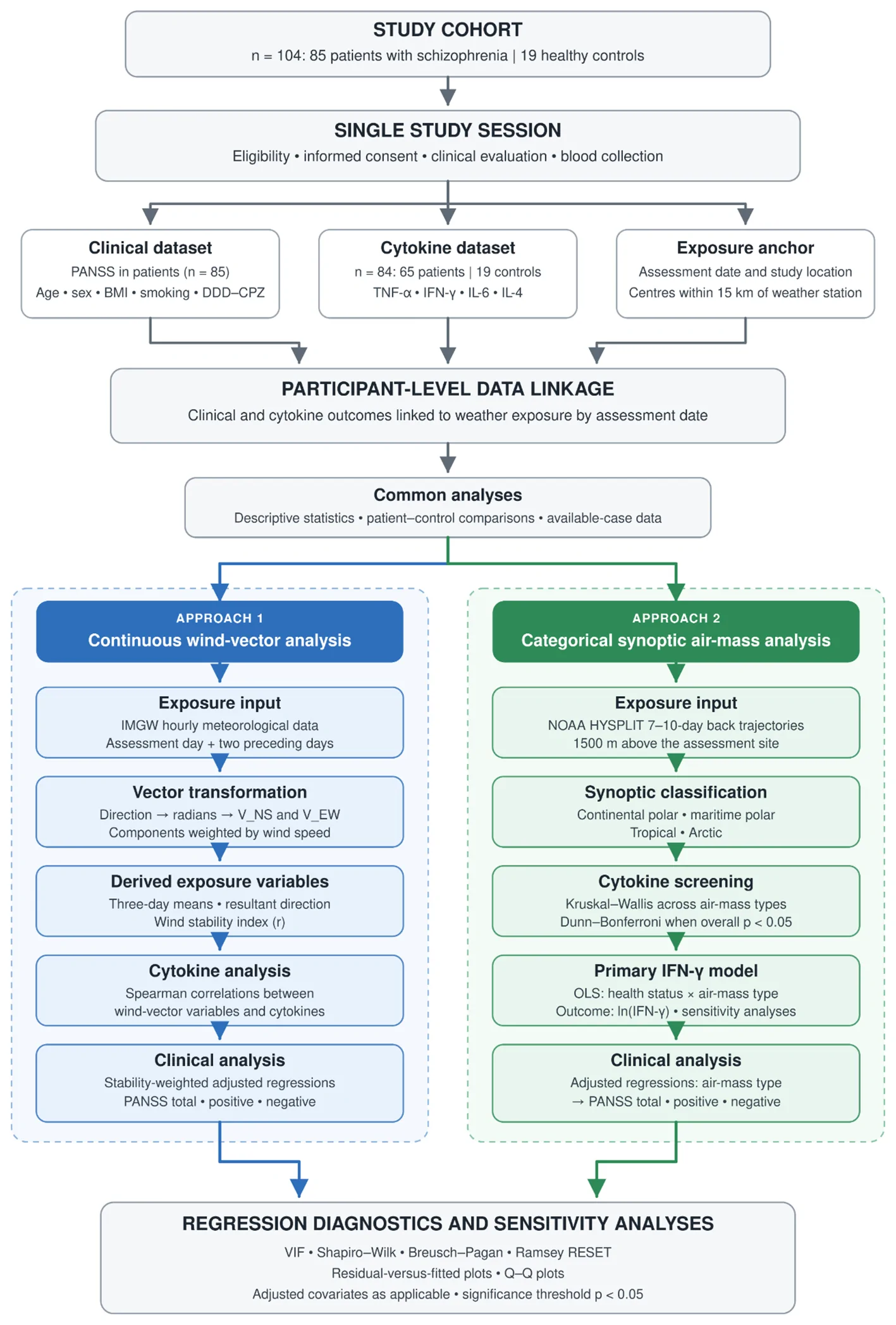
Flowchart and study design. The analysis was based on available data. When data were unavailable, the number of observations included in the analysis was reduced accordingly.

### 2.1 Participants

Patients were recruited from three clinical centers located within a 15 km radius of the meteorological station. All centers are situated in the Podlaskie Voivodeship, Poland, a region characterized by pronounced seasonal variation throughout the year. Exclusion criteria included: inability to provide informed consent; exposure to acute stressors or major life events within the preceding 8 weeks; alcohol or psychoactive substance abuse; recent changes (within 4 weeks) in psychiatric or anti-inflammatory treatment; intensive physical activity within the preceding 72 hours; the presence of autoimmune disorders, Human Immunodeficiency Virus (HIV) infection, or viral hepatitis; other severe somatic illnesses that could affect biochemical measurements; recent travel to different climate zones; shift work disrupting the natural sleep-wake rhythm; and insufficient exposure to outdoor weather conditions (defined as less than 1 hour).

The study included patients with schizophrenia and a disease duration of at least several years: first-episode, drug-naïve patients were excluded. Eligible participants were aged between 18 and 65 years. Participant characteristics are presented in **Table 1**.

**Table 1:** Demographic, clinical, immunological, and meteorological characteristics of the study population.

Parameter	Total sample (n=104)	Schizophrenia patients (n = 85)	Healthy controls (n = 19)	*p*-value
Demographics				
Age, years	38.5 [31.0–49.5]	40.0 [32.0–50.0]	40.0 [32.0–50.0]	0.081^*^
Sex [n, % men]	67 (64.4)	56 (65.9)	11 (57.9)	0.511^*^
BMI, kg/m^2^	26.8 [23.45–30.00]	26.6 [23.3–31.3]	27.6 [24.2–29.9]	0.918^*^
Smoking [n, %]	62 (59.6)	53 (62.4)	9 (47.4)	0.229^**^
Clinic - patients only				
Equivalent CPZ [mg/d]	–	700[450–900]	–	–
PANSS - Total P	–	19 [15–26]	–	–
PANSS - Total N	–	20 [14–26]	–	–
PANSS general	–	42 [36–48]	–	–
PANSS - Total amount	–	82[69–92]	–	–
Meteorology				
Mean temperature, °C	7.6 [2.37–13.39]	8.3 [2.37–14.40]	7.6 [2.37–8.30]	0.680^*^
Relative humidity, %	75.33 [68.28–82.86]	71.53 [61.70–82.46]	82.86 [71.53–91.88]	0.003^*^
Atmospheric pressure, hPa	1000.11 [996.70–1001.86]	999.11 [996.70–1001.86]	1000.11 [996.34–1001.86]	0.574^*^
Wind stability (*r*)	0.660 [0.360–0.817]	0.660 [0.360–0.854]	.659 [0.070–0.700]	0.163^*^
Cytokines subsample, N	84	65	19	
TNF-α	135.45 [118.98–151.26]	136.56 [121.77–151.26]	128.76 [118.05–144.34]	0.356^*^
IFN-γ	42.62 [33.81–53.20]	44.38 [38.74–55.66]	26.04 [17.56–41.56]	<0.001^*^
IL-6	1.95 [1.16–3.50]	2.34 [1.55–3.89]	1.20 [0.80–2.34]	0.004^*^
IL-4	0.67 [0.52–0.92]	0.62 [0.62–0.82]	0.81 [0.40–1.07]	0.900^*^
Air mass/wind type distribution, n (%)				
Polar Continental Air	17 (16.4)	17 (20.0)	0 (0.0)	**0**.022^***^
Polar Maritime Air	43 (41.3)	36 (42.4)	7 (36.8)	
Arctic Air	23 (22.1)	19 (22.4)	4 (21.1)	
Tropical Air	21 (20.2)	13 (15.3)	8 (42.1)	

Continuous variables are presented as median [interquartile range], and categorical variables as n (%). ^*^Mann-Whitney U test, ^**^chi-square test, ^***^Fisher’s exact test. CPZ, Chlorpromazine equivalent; PANSS, Positive and Negative Syndrome Scale.

Bold values indicate statistically significant p-values (p < 0.05).

### 2.2 Procedures

Patients were assessed by clinicians using the Positive and Negative Syndrome Scale (PANSS) (22). During the psychiatric interview and clinical examination, eligibility for study inclusion was determined and written informed consent was obtained. Medication doses were included in the analyses using defined daily dose chlorpromazine equivalents (DDD-CPZ) (23). Body mass index (BMI, kg/m^2^), smoking status (yes: current or past smoking; no), age, and sex (1 – male) were included as covariates.

Serum was separated within 1 h of collection, stored at −80 °C, thawed for 12 hours at −20 °C and then for 6 hours at 4 °C, and analyzed using the Bio-Plex Pro™ Human Cytokine Screening Panel, 48-Plex (Bio-Rad, Cat. No. 12007283), on a Bio-Plex 200 system. Although the panel measured 48 immune mediators, IFN-γ, TNF-α, IL-6, and IL-4 were selected *a priori* for the present study, before inspection of the assay results, based on the prespecified inflammatory and Th1/Th2-related hypotheses. Samples were diluted 1:4 and analyzed in single wells; nine-point standards, blanks, and kit controls were run in duplicate. Measurements were performed on two consecutive days using the same instrument and standard lot but different assay lots, with separate blank-corrected five-parameter logistic calibration curves fitted for each plate. Dilution-adjusted operational LLOQs were 7.65-7.69 pg/mL for IFN-γ, 0.16-0.23 pg/mL for IL-4, 0.37-8.00 pg/mL for IL-6, and 6.47-10.46 pg/mL for TNF-α. Median CVs for accepted duplicate standards were 0.69–4.52%, and control-well CVs were 1.49-21.76%. Sample-level intra- and inter-assay CVs could not be calculated because samples were measured in single wells and no common serum sample was repeated across plates; therefore, residual batch effects cannot be excluded.

Meteorological data from the nearest standardized weather station were obtained from official public sources provided by the Institute of Meteorology and Water Management (IMGW), in accordance with the Act of August 11, 2021, on open data and the reuse of public sector information.

### 2.3 Meteorological data processing

Meteorological data (wind speed (m/s), mean air temperature (°C), station-level atmospheric pressure (hPa), and relative humidity (%)) were obtained from hourly records provided by the Institute of Meteorology and Water Management (IMGW) from the station located closest to the study site, within a radius of up to 15 km. Three-day averages were calculated, including the day of assessment and the two preceding days. This time window was selected to account for the expected delay in immunological and clinical responses to specific weather conditions. The rationale for applying this time lag was based on the concept of immunoseasonality, which assumes exposure to common environmental antigens whose occurrence varies with weather conditions (15, 24). However, this weather-exposure window may have been insufficient (25); therefore, as a sensitivity analysis, we additionally examined variables averaged over 7 days (**Supplementary Table S1B**).

Our study approached air mass exposure as a potential marker of immunological and clinical status using two distinct conceptual strategies. The first approach was based on a trigonometric transformation of wind direction, adjusted for air mass inflow by wind speed and the stability of inflow direction, represented by the wind stability index described below. This transformation was used to obtain more precise information on wind flow along the north–south and east–west axes. This approach allowed wind exposure to be analysed as a continuous variable, without substantial loss of information across statistical models. However, it is relatively non-specific. For a given wind direction, it does not distinguish the specific physical characteristics carried by the air mass, such as dryness, humidity, or typical changes in atmospheric pressure and temperature.

The second conceptual approach was based on a categorical variable, in which air masses during the analyzed time period were classified according to their source region and typical meteorological characteristics for the Podlasie region. These categories included continental polar air masses, maritime polar air masses, tropical air masses, and Arctic air masses.

#### 2.3.1 Continuous wind-vector approach based on trigonometric decomposition

Due to the circular nature of wind direction, raw angular data expressed in degrees could not be directly included in statistical analyses. Therefore, all calculations were initially performed on hourly data. To avoid mathematical artifacts associated with arithmetic averaging of angular measures, trigonometric decomposition was applied. Wind direction values were first converted from degrees to radians. Each hourly wind direction measurement was then decomposed into two orthogonal vector components: a sine component representing the East-West axis and a cosine component representing the North-South axis.

To reflect the actual transport of air masses from a given direction, wind speed was incorporated into both vector components. This procedure yielded:

Meridional (North–South) component:


$$V_{NS}=v\cdot \text{cos}(\theta )$$

where *v* denotes wind speed and θ wind direction expressed in radians; positive values indicate northerly winds, while negative values indicate southerly winds.

Zonal (East–West) component:


$$V_{EW}=v\cdot \text{sin}(\theta )$$

where positive values indicate easterly winds and negative values indicate westerly winds.

For each participant, mean values of both vector components were calculated over the three-day period and seven-day period (for sensitivity analyses **Supplementary Table S2B**), resulting in continuous predictors representing the dominant direction and strength of atmospheric transport during the exposure window. After calculations based on vector components, a back-transformation (recomposition) was performed to obtain the final mean wind direction for each observation period. The mean wind direction was calculated using the arctangent function with quadrant correction (ATAN2) according to the formula:


$$Mean\;wind\;direction=mod(atan2(V_{EW},V_{NS})\cdot \frac{180}{\pi }+360,\;360)$$

This approach enabled recovery of the resultant wind vector angle in a 0–360°format, allowing for a physically meaningful interpretation of the dominant direction of air mass inflow while accounting for wind speed in each case.

Additionally, a wind stability index (*r*) was calculated based on the same three-day period, quantifying the degree of consistency in wind direction during the observation window. The index ranges from 0 to 1, with values closer to 1 indicating a stable and persistent air mass inflow from a single direction, and values closer to 0 reflecting high directional variability and instability of wind flow. The index was calculated according to the following formula:


$$r=\frac{\sqrt{{\bar{V}}_{NS}^{\;2}+{\bar{V}}_{EW}^{\;2}}}{{\bar{v}}_{\text{speed}}}$$

where ${\bar{V}}_{NS}$and ${\bar{V}}_{EW}$represent the mean meridional and zonal vector components, respectively, and ${\bar{v}}_{\text{speed}}$denotes the arithmetic mean wind speed over the three-day period.

#### 2.3.2 Categorical synoptic air-mass approach

Air mass origin was assessed using the NOAA HYSPLIT trajectory model (26, 27) for archive data. The period from 7 days prior to the patient assessment was evaluated in order to determine the origin and trajectories of air masses at an altitude of 1500 meters over the patient assessment site. The trajectories were assessed visually, and based on this evaluation, air masses were classified as follows: Continental Polar air masses, Maritime Polar air masses, Tropical air masses, and Arctic air masses. Arctic Air originates from the Arctic region and is generally associated with cold conditions and marked decreases in temperature. Polar Continental Air typically advects from eastern Europe and Asia and is characterized by low humidity; in Poland, it is usually associated with dry and cold conditions in winter, and dry, hot conditions in summer. Polar Maritime Air originates over the North Atlantic and is typically humid, often bringing increased cloud cover, precipitation, and higher atmospheric moisture. Tropical Air was used as an operational category for air masses advecting from the south; in the Polish climatic context, such air masses are usually associated with higher temperatures and, depending on their maritime or continental origin, either humid convective conditions or dry, sunny weather. These categories are consistent with standard meteorological descriptions of air masses affecting Poland (28) (**Supplementary Figure S9**). For each assessment date, a backward air-mass trajectory simulation was generated for the predefined study location (53.1°N, 22.9°E; 125 m above sea level). Air mass classification was based on visual inspection of the trajectory, including its overall direction of advection, geographical origin, intermediate positions at 24-hour intervals, and changes in trajectory altitude over the analyzed period. The trajectory information was additionally interpreted in the context of the corresponding meteorological conditions, including the average temperature, relative humidity, and atmospheric pressure described above. Each trajectory was independently classified by two researchers according to the predefined air mass categories. Any disagreement between the two raters was resolved by assessment by a third researcher (initial inter-rater agreement: 87.5%; Cohen’s κ = 0.819, 95% CI [0.705, 0.934], p < 0.001). During trajectory classification, the researchers were blinded to the participants’ clinical status.

### 2.4 Statistical analysis

Data analyses were performed using Microsoft Excel and StataNow 19 SE. Analyses were conducted using available-case data. The total cohort included 104 participants, whereas cytokine analyses were performed in a subsample of 84 participants. Between-group comparisons were performed using the Mann-Whitney U test for continuous variables and Pearson’s chi-square test or Fisher’s exact test for categorical variables, as appropriate. Continuous variables were presented as medians and interquartile ranges, whereas categorical variables were presented as counts and percentages (**Table 1**).

After preparing the meteorological variables described above, a series of statistical analyses was performed. For the continuous wind-vector variables, Spearman correlation analyses were first conducted to characterize the meteorological information carried by each vector component. These variables were also correlated with cytokine concentrations to explore potential associations between wind inflow direction and peripheral immune markers. Next, the associations between wind-vector components and clinical status, expressed by PANSS scores, were assessed using linear regression models. These models were weighted by the wind direction stability index, *r*, to account for the consistency of wind inflow direction during the exposure window. The models were adjusted for age, sex, BMI, smoking status, and chlorpromazine equivalent dose.

For the categorical synoptic air-mass approach, cytokine concentrations were first compared across air mass types using the Kruskal-Wallis test. For cytokines showing significant overall differences, Dunn-Bonferroni *post-hoc* tests were performed. Subsequently, after identifying IFN-γ as a cytokine of interest, an OLS linear regression model with an interaction term was fitted to assess whether the association between air mass type and ln(IFN-γ) differed between patients with schizophrenia and healthy controls. IFN-γ concentrations were natural-log-transformed before inclusion in linear regression models to reduce skewness and improve model assumptions regarding residual distribution and variance homogeneity. The model included health status, air mass type, and their interaction as categorical predictors. Sensitivity analyses were also performed by excluding air mass type 1, for which no healthy controls were available, and by additionally adjusting the model for age, sex, BMI, smoking status, seasons and mean temperature.

To evaluate whether synoptic air mass type was directly associated with clinical symptom severity, additional linear regression models were fitted with PANSS total, positive, and negative scores as dependent variables. These models included air mass type as the main predictor and were adjusted for age, sex, BMI, smoking status, and chlorpromazine equivalent dose. Between-group differences in cytokine concentrations between patients with schizophrenia and healthy controls were assessed using Mann-Whitney U tests.

The threshold for statistical significance was set at p < 0.05. Regression model diagnostics were also performed. These included assessment of multicollinearity using variance inflation factors, with values below 3 considered acceptable, the Shapiro-Wilk W test for normality of residuals, the Breusch-Pagan test for homoscedasticity, and the Ramsey RESET test for model specification. In addition, residuals-versus-fitted plots and quantile-quantile plots were visually inspected. Residual diagnostics indicated potential heteroskedasticity across the small and unbalanced health-status × air-mass cells. Therefore, all reported interaction and sensitivity models were estimated using ordinary least squares with HC3 heteroskedasticity-consistent standard errors, which provide greater protection against heteroskedasticity and high-leverage observations in finite samples. Regression coefficients and estimated marginal means were based on the OLS estimates, whereas standard errors, confidence intervals, and p-values were calculated using the HC3 variance estimator.

## 3 Results

### 3.1 Local wind-vector decomposition approach

#### 3.1.1 Characteristics of winds in Spearman correlations

Spearman correlations between the vectors components and meteorological variables showed statistically significant associations only with relative humidity (ρ = −.458, p < 0.001 for $V_{NS}$; ρ = .398, p < 0.001 for $V_{EW}$), indicating that, within the vector-based approach, southerly and easterly flows tended to be more humid than northerly and westerly flows (**Supplementary Table S1**).

#### 3.1.2 Correlations between wind components and cytokines

In exploratory correlation analyses, northerly winds were positively correlated with TNF-α (ρ = .351, p < 0.001). Westerly winds were correlated with TNF-α (ρ = −.322, p = 0.003), while IFN-γ shows a trend close to statistical significance (ρ = −.194, p =0.078)(**Supplementary Table S2**).

#### 3.1.3 Regression models

Results of the weighted linear regression analyses examining the association between wind direction and PANSS scores are presented in **Supplementary Table S3**. Neither $V_{NS}$ or $V_{EW}$ was significantly associated with PANSS scores. Across the models, CPZ-equivalent dose was the only consistent significant predictor of clinical status (for PANSS total: B = 0.016; 95% CI [0.008, 0.025]; p <0.001), while smoking (B = 4.09; 95% CI [0.292, 7.891]; p =0.035) and male sex (B = −4.93; 95% CI [−8.98, −0.884]; p = 0.018) were additionally significant predictors in the model for negative symptoms. All models demonstrated acceptable fit, with overall model significance.

### 3.2 Categorical synoptic air-mass approach

#### 3.2.1 Cytokine profiles across synoptic air mass types

Differences in cytokine concentrations across air mass types, assessed using the Kruskal–Wallis test, are presented in **Table 2**. Statistically significant overall differences across air-mass types were observed for IFN-γ (Kruskal–Wallis χ² (3) = 10.332, p = 0.016) and TNF-α (χ² (3) = 17.213, p = 0.001). Dunn–Bonferroni *post-hoc* comparisons showed that concentrations under Tropical-air masses were significantly lower than under Polar Continental, Polar Maritime, and Arctic masses for both IFN-γ (median [IQR], 37.33 [33.10–41.56]; adjusted p = 0.045,.013, and 0.021, respectively) and TNF-α (106.87 [82.41–132.87]; adjusted p = 0.004, 0.013, and 0.001, respectively). No significant differences across air-mass types were observed for IL-6 (χ² (3) = 2.771, p = 0.429) or IL-4 (χ² (3) = 5.783, p = 0.123).

**Table 2:** Cytokine concentrations according to air mass type in patients with schizophrenia, N = 65.

Cytokine	Polar Continental Air	Polar Maritime Air	Arctic Air	Tropical Air	Kruskal-Wallis χ²	*p*-value	*Post-hoc* comparisons
IFN-γ	52.84 [42.97–55.66]	45.79 [41.56–57.07]	46.85 [38.39–66.95]	37.33 [33.10–41.56]	10.332	**0**.**016**	Tropical Air < Polar Continental Air, p = 0.045; Tropical Air < Polar Maritime Air, p = 0.013; Tropical Air < Arctic Air, p = 0.021
TNF-α	153.09 [149.43–191.31]	136.56 [125.47–151.26]	145.97 [131.02–169.53]	106.87 [82.41–132.87]	17.213	**0**.**001**	Tropical Air < Polar Continental Air, p = 0.004; Tropical Air < Polar Maritime Air, p = 0.013; Tropical Air < Arctic Air, p = 0.001
IL-6	3.12 [1.95–3.12]	2.14 [1.55–3.12]	3.12 [1.75–5.25]	1.75 [1.16–3.50]	2.771	0.429	—
IL-4	0.92 [0.82–0.92]	0.62 [0.51–0.82]	0.82 [0.62–0.87]	0.62 [0.40–1.01]	5.783	0.123	—

Values are presented as median [interquartile range]. Between-group comparisons across air mass types were performed using the Kruskal-Wallis test. For significant Kruskal-Wallis results, Dunn-Bonferroni *post-hoc* comparisons were performed. *Post-hoc* p-values are Bonferroni-adjusted. Only significant *post-hoc* comparisons are shown in the main table; all remaining pairwise comparisons were non-significant after Bonferroni correction. Results involving type 1 air masses should be interpreted cautiously due to the small number of observations in this category. IFN-γ , interferon gamma; TNF-α , tumor necrosis factor alpha; IL , interleukin.

Bold values indicate statistically significant p-values (p < 0.05).

#### 3.2.2 Health-status-dependent IFN-γ patterns across air mass types

IFN-γ was selected for further analyses because it is a key Th1-associated pro-inflammatory cytokine frequently investigated as a marker of immune seasonality. The interaction analysis specifically examined whether IFN-γ concentrations varied differently across air mass types in patients with schizophrenia and healthy controls. The interaction model was significant (N = 84, R² = .520, adjusted R² = .483, p < 0.001; **Supplementary Table S4**). Among patients, Tropical air masses was associated with lower ln(IFN-γ) than Polar Maritime air masses (B = −0.235, 95% CI [−0.357, −0.112], p < 0.001) and Arctic air masses (B = −0.273, 95% CI [−0.492, −0.054], p = 0.015). Conversely, among healthy controls, Tropical air masses was associated with higher ln(IFN-γ) than Polar Maritime air masses (B = 0.697, 95% CI [0.233, 1.161], p = 0.004) and Arctic air (B = 0.879, 95% CI [0.554, 1.205], p < 0.001). The corresponding interaction contrasts were significant for both Tropical versus Polar Maritime air masses and Tropical versus Arctic air masses, confirming directionally opposite patterns in patients and controls (**Table 3**).

**Table 3:** Planned contrasts comparing tropical air with polar maritime and Arctic air in the health-status × air-mass interaction model for ln(IFN-γ).

Comparison	Patients	Healthy controls	Interaction contrast
Tropical Air vs Polar Maritime Air	−0.235 [−0.357, −0.112]; 0.001	0.697 [0.233, 1.161]; 0.004	0.932 [0.452, 1.412]; <0.001
Tropical Air vs Arctic Air	−0.273 [−0.492, −0.054]; 0.015	0.879 [0.554, 1.205]; <0.001	1.152 [0.76, 1.545]; <0.001

Values are presented as unstandardized contrasts, HC3 heteroskedasticity-consistent 95% confidence intervals, and p-values. Estimates for patients and healthy controls represent within-group differences in ln(IFN-γ), calculated as Tropical Air minus the respective comparison air mass. The interaction contrast represents the difference between these group-specific contrasts (healthy controls minus patients) and tests whether the association between air mass type and ln(IFN-γ) differs by health status. Comparisons involving Polar Continental Air were not included because no healthy controls were observed under this air mass type.

Healthy controls had lower estimated ln(IFN-γ) than patients under Polar Maritime air masses (B = −0.812, 95% CI [−1.171, −0.452], p < 0.001) and Arctic air masses (B = −1.033, 95% CI [−1.263, −0.802], p < 0.001). No significant between-group difference was observed under Tropical air masses (B = 0.120, 95% CI [−0.198, 0.438], p = 0.454; **Supplementary Tables S5**, **S6**). These findings remained stable after excluding Polar Continental air masses and after adjustment for seasonality, mean temperature, age, sex, BMI, and smoking status (**Supplementary Tables S7**, **S8**).

#### 3.2.3 Synoptic air mass type and PANSS symptom severity

No air mass type was a significant predictor of patients’ clinical status as measured by PANSS scores. Across the models, only CPZ-equivalent dose was significantly associated with PANSS scores (B = 0.018, 95% CI [0.009, 0.027]; p < 0.001). Furthermore, the model predicting negative symptoms did not reach statistical significance. The results are presented in **Table 4**.

**Table 4:** Linear regression models predicting PANSS scores according to air mass type.

Predictor	PANSS total	PANSS positive	PANSS negative
Polar Continental Air vs Polar Maritime Air	2.18 [−8.26, 12.61]; 0.679	0.34 [−3.86, 4.54]; 0.873	1.57 [−3.06, 6.20]; 0.501
Arctic Air vs Polar Maritime Air	3.87 [−6.37, 14.10]; 0.454	1.36 [−2.76, 5.48]; 0.512	1.97 [−2.57, 6.52]; 0.390
Tropical Air vs Polar Maritime Air	2.22 [−9.30, 13.74]; 0.702	0.79 [−3.84, 5.43]; 0.734	0.44 [−4.68, 5.55]; 0.866
Age, years	−0.25 [−0.60, 0.11]; 0.166	−0.02 [−0.16, 0.12]; 0.809	−0.09 [−0.25, 0.07]; 0.256
Sex, male = 1	−3.21 [−12.25, 5.82]; 0.481	1.95 [−1.68, 5.59]; 0.288	−2.74 [−6.75, 1.28]; 0.178
BMI, kg/m²	−0.28 [−0.95, 0.39]; 0.405	−0.04 [−0.31, 0.23]; 0.764	−0.10 [−0.40, 0.19]; 0.487
Smoking, 1 = yes	−3.26 [−11.91, 5.40]; 0.456	−2.89 [−6.37, 0.59]; 0.102	3.23 [−0.61, 7.08]; 0.098
CPZ equivalent, mg/day	0.018 [0.009, 0.027]; < 0.001	0.006 [0.003, 0.010]; 0.001	0.004 [0.0004, 0.008]; 0.030
Constant	87.73 [62.30, 113.17]; < 0.001	16.26 [6.03, 26.49]; 0.002	22.35 [11.06, 33.65]; < 0.001
Model statistics	N = 85; R² = 0.222; Adj. R² = 0.140; F (8,76) = 2.71; p = 0.011	N = 85; R² = 0.185; Adj. R² = 0.100; F (8,76) = 2.16; p = 0.040	N = 85; R² = 0.115; Adj. R² = 0.022; F (8,76) = 1.24; p = 0.288

Reference category: Polar Maritime Air.

Values are presented as unstandardized regression coefficients, 95% confidence intervals, and p-values. Air mass type 2 was used as the reference category. Bolded values indicate statistical significance at p < 0.05. CPZ, chlorpromazine equivalent; PANSS, Positive and Negative Syndrome Scale; BMI, body mass index; CI, confidence interval.

## 4 Discussion

### 4.1 Main finding (Categorical synoptic air-mass approach)

The principal finding of our study was the health-status-dependent association between synoptic air mass type and IFN-γ concentrations. Patients with schizophrenia and healthy controls exhibited directionally opposite IFN-γ patterns across air mass types (**Figure 2**). Among patients, Tropical air masses were associated with lower ln(IFN-γ) than Polar Maritime and Arctic air masses. Conversely, among healthy controls, Tropical air masses were associated with higher ln(IFN-γ) than both Polar Maritime and Arctic air masses. The significant interaction contrasts confirmed that these patterns differed between the groups rather than merely reflecting an overall difference in IFN-γ concentrations (**Table 3**). Accordingly, the marked patient-control differences observed under Polar Maritime and Arctic air masses were no longer present under Tropical air masses. Importantly, this interaction remained stable after excluding polar continental air, for which no control observations were available, and after adjustment for seasons, temperature, age, sex, BMI, and smoking status (**Supplementary Tables S4**–**S10**).

**Figure 2:**
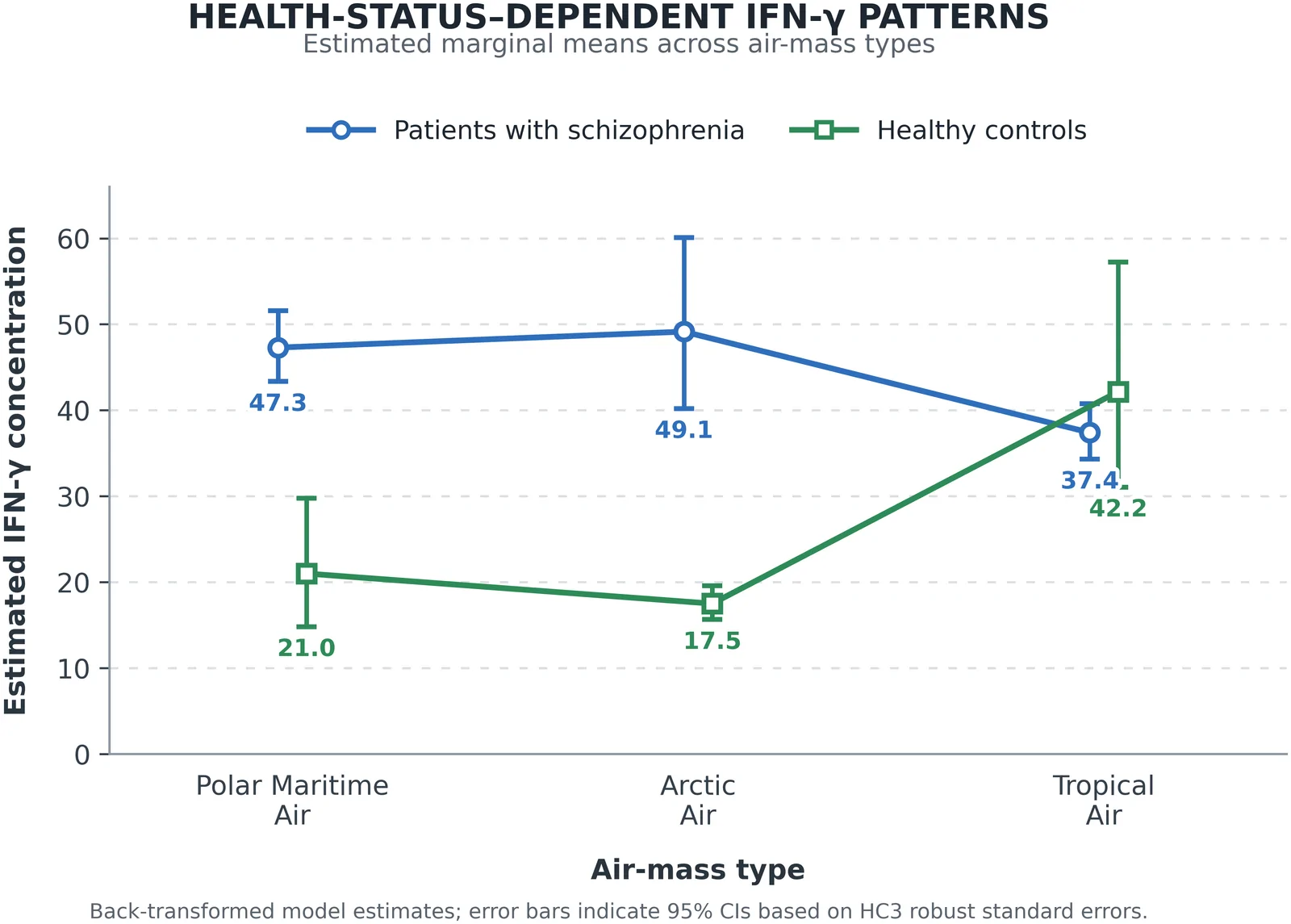
Predicted IFN-γ concentration (pg/mL). Points represent back-transformed estimated marginal means, and error bars indicate 95% confidence intervals based on HC3 robust standard errors. Cell sizes for the displayed estimates were: Polar Maritime air masses - patients with schizophrenia, n = 33, and healthy controls, n = 7; Arctic air masses - patients, n = 16, and controls, n = 4; and Tropical air masses - patients, n = 11, and controls, n = 8. Polar Continental air masses was not displayed because no healthy controls were observed under this condition (patients, n = 5; controls, n = 0).

A second important finding was the distinct cytokine profile associated with Tropical air masses among patients with schizophrenia. In this group, Tropical air masses were associated with lower concentrations of both IFN-γ and TNF-α than each of the other air mass types (**Table 2**). The convergence of findings for two Th1-associated or pro-inflammatory cytokines suggests that Tropical air masses may be associated with a broader alteration in immune activity among patients. However, this pattern should not be interpreted as generalized immunosuppression because no corresponding differences were found for IL-6 or IL-4, and the health-status interaction was examined only for IFN-γ.

Third, patients with schizophrenia exhibited higher overall IFN-γ concentrations than healthy controls (**Table 1**). Although altered IFN-γ signaling in schizophrenia is not itself unexpected, its importance in the present study lies in the observation that this between-group difference was not uniform across environmental conditions. Instead, it was pronounced under Polar maritime and Arctic air masses but absent under Tropical air masses. Thus, the findings indicate not only an overall difference in inflammatory activity but, more importantly, a different pattern of immune responsiveness to synoptic environmental conditions.

The concept of immunoseasonality may provide a framework for interpreting this pattern (10, 15). This theory assumes that seasonal environmental signals - including temperature, photoperiod, humidity, circulating pathogens, aeroallergens, and other meteorological exposures - contribute to periodic shifts in immune activity. Colder periods, reduced sunlight exposure, and increased circulation of viral pathogens may favor cell-mediated and Th1-associated immune responses, including IFN-γ production (29–31). Conversely, warmer periods often coincide with increased exposure to pollen and other aeroallergens, which may promote humoral and Th2-associated responses in sensitized individuals (32, 33). These responses are not mutually exclusive, but their relative balance may change depending on the environmental context. Within this framework, the higher IFN-γ concentrations observed in patients under non-tropical air masses may be compatible with enhanced Th1-associated activity under synoptic conditions more commonly associated with cooler parts of the year (34, 35). In patients with schizophrenia, who may already exhibit baseline immune dysregulation (8, 36), such activation could be stronger, more persistent, or less effectively regulated than in healthy individuals (37). This interpretation may help explain why the largest patient-control differences were observed under Polar Maritime and Arctic air masses. Tropical air masses, by contrast, generally bring warmer conditions and occur more frequently during the warmer part of the year, although they may be either dry or humid depending on their origin and trajectory. Within the immunoseasonal framework, warmer conditions and seasonal aeroallergen exposure could promote a relative shift away from Th1-associated signaling and toward humoral or Th2-related activity in susceptible individuals (15, 38). The lower IFN-γ observed among patients under tropical air may therefore reflect a context-dependent reduction in Th1-associated activity rather than a general normalization of immune function. The opposite pattern observed in healthy controls is particularly noteworthy. Experimental studies in healthy volunteers have shown that experimentally induced fever-range whole-body hyperthermia may enhance the capacity of peripheral lymphocytes and T cells to produce IFN-γ following ex vivo stimulation (39, 40). This short-term enhancement of IFN-γ responsiveness may reflect an adaptive physiological response to thermal stress. However, experimentally induced hyperthermia is not directly equivalent to exposure to warmer ambient conditions or tropical air masses (30). Whether patients with schizophrenia exhibit comparable immunological flexibility remains unknown. Nevertheless, evidence of cytokine-network dysregulation and possible regulatory T-cell dysfunction in schizophrenia provides a rationale for hypothesizing that their responses to environmental transitions may involve a different balance of regulatory, Th1-, and Th2-related (36, 41). Consequently, tropical air may be associated with increased IFN-γ responsiveness in healthy individuals but reduced IFN-γ signaling in patients with pre-existing immune dysregulation. This explanation remains hypothetical but is consistent with the directionally opposite patterns identified by the interaction model. The potential relevance of these immune shifts extends beyond peripheral cytokine concentrations. Peripheral inflammatory signals can communicate with the central nervous system through several pathways involving endothelial and immune cells at the blood-brain barrier, active transport mechanisms, circumventricular structures, and neural signaling pathways (42–46). Such immune-to-brain communication may affect microglial activity, the hypothalamic-pituitary-adrenal axis (47, 48), and dopaminergic or glutamatergic neurotransmission (48), thereby potentially influencing vulnerability to psychosis (49, 50). The possible clinical relevance of warmer synoptic conditions is also supported by retrospective epidemiological observations. Warmer periods have been associated with increased disinhibition, impulsivity, risk-taking behavior, aggression, and affective activation (16, 51–54). Some studies have additionally identified seasonal or temperature-related increases in hospitalizations due to psychotic exacerbations. These observations do not demonstrate that IFN-γ mediates the association between summer conditions and psychosis. Nevertheless, they suggest that the warmer environmental context accompanying Tropical air masses may coincide with a period of increased vulnerability to behavioral activation and psychotic exacerbation. Within the immunoseasonal theory, a maladaptive shift in immune balance could represent one of several biological mechanisms contributing to this vulnerability (15).

The third finding is that air mass classification may be particularly useful because it represents a combination of temperature, humidity, pressure, atmospheric trajectory, and other co-occurring environmental characteristics rather than an isolated meteorological parameter - the complete interaction model explained approximately 52% of the variance in ln(IFN-γ). This proportion reflects the joint contribution of health status, air mass type, and their interaction and should not be attributed to air mass type alone. Nevertheless, the model indicates that immune status may be strongly associated with the combination of environmental conditions and underlying health status.

The fourth main finding was the absence of a direct association between either categorical air mass type or continuous wind-vector components and PANSS symptom severity (**Table 4**; **Supplementary Table S3**). This suggests that the observed immunological patterns were not simply a reflection of concurrent differences in clinical symptom severity. However, the absence of a direct association with PANSS does not exclude an indirect pathway in which environmental conditions modulate immune activity and thereby contribute to longer-term vulnerability to symptom exacerbation or psychosis (21). Such a pathway was not directly tested in the present study and should therefore be considered a hypothesis for future longitudinal research.

### 4.2 Other findings

#### 4.2.1 Local wind-vector decomposition approach

The wind-vector regression models did not reveal significant associations with PANSS scores. Spearman correlations suggested that, despite incorporating wind persistence, wind speed, and multi-day averaging, the continuous vector approach captured a narrower range of meteorological information than the synoptic model: among the measured meteorological variables, the vector components showed only moderate correlations with relative humidity. In the sensitivity analysis, however, the 7-day wind-vector components showed substantially stronger and more consistent correlations with temperature, relative humidity, and atmospheric pressure. This pattern suggests that the shorter, 3-day exposure window may be more sensitive to transient meteorological fluctuations, whereas averaging wind-vector components over 7 days may reduce the contribution of short-term variability and better capture persistent meteorological conditions. Thus, longer averaging windows may allow continuous wind-vector measures to serve as more informative proxies for the broader meteorological environment. What is more, extending the averaging window did not substantially alter the cytokine-related pattern, as the direction and magnitude of the associations between the wind-vector components and TNF-α were closely reproduced in the 7-day analysis.

#### 4.2.2 Regression models

As noted above, neither the wind-vector components nor air mass types were significantly associated with PANSS outcomes in the regression models; nevertheless, the analyses yielded several additional findings (**Supplementary Table S3**; **Table 4**). Interestingly, medication dose was a significant predictor across all models, with the strongest effects observed for positive symptoms, which may have a straightforward explanation: more symptomatic patients receive higher doses of antipsychotic medication, while psychotic symptoms, as measured by the PANSS positive subscale, are often particularly important in decisions to increase the dose. Additionally, smoking emerged as a variable exerting opposing effects on symptom domains: smoking was associated with lower severity of positive symptoms and higher severity of negative symptoms in vectors models. One possible explanation for this apparent paradox is the well-described nicotine self-medication hypothesis (55), and the regression results may represent an example of patients’ attempts at self-medication in response to increasing difficulties within the domain of negative symptoms.

### 4.3 Strengths, limitations, and future directions

Our study has several strengths. One is its novel integration of clinical, cytokine, and synoptic data using two complementary approaches to characterize environmental exposure: a continuous wind-vector model and a trajectory-based synoptic air-mass classification. The weighted analyses incorporated a wind-stability index, while the vector model additionally accounted for wind speed. We used data from official and reliable sources and included a small but exploratorily informative group of healthy controls. Furthermore, the relatively strict eligibility criteria, which partly contributed to the limited sample size, helped reduce potential confounding and sample heterogeneity. The use of model diagnostics, sensitivity analyses, and HC3 heteroskedasticity-consistent standard errors further supported the robustness of the IFN-γ interaction finding.

The study also has several important limitations. Its exploratory, pilot, and cross-sectional design permits the identification of associations but does not establish their temporal direction or causality. Each participant underwent only a single assessment; repeated cytokine measurements accompanied by clinical evaluations would allow more precise conclusions regarding within-person changes. The study groups were small and unbalanced and may not fully represent the broader population. Although model diagnostics and sensitivity analyses were applied to address these concerns, they cannot completely eliminate the associated uncertainty. Moreover, the sample was recruited from a specific region of Poland - the Podlaskie Voivodeship - which limits the generalizability of the findings. Larger, longitudinal, multicenter studies are therefore required. Another limitation concerns antipsychotic medication dosage. In our models, we controlled only for the overall antipsychotic dose, without distinguishing between first- and second-generation antipsychotics. This is particularly relevant given the differences in adverse-effect profiles between these drug classes, which may themselves influence some of the clinical manifestations of schizophrenia. An additional limitation is that the present study represents a reanalysis of previously collected data, and the analyzed sample largely overlaps with that used in our previous study. Therefore, the current findings should be interpreted as an extension of earlier analyses rather than as evidence derived from an entirely independent cohort. Additionally, the choice of 3-day and 7-day averaging windows for meteorological exposures remains only partially justified, as the timing of immune responses may differ depending on the specific infectious agent, antigen, or immunological pathway involved (56, 57), particularly given the distinct temporal dynamics of cytokine responses within the central nervous system (CNS) (58). A further limitation of using averaged exposures is the potential loss of information on the contribution of individual days to immune activation. At the same time, multi-day averages provide a pragmatic way to summarize delayed exposure effects without introducing multiple, highly overlapping lagged predictors into the same model (59). Future studies should therefore define exposure windows more specifically according to the immunological pathway under investigation and, where possible, apply lag-based approaches extending up to approximately 10 days to better characterize the temporal relationship between meteorological exposure, immune activation, and subsequent behavioral or clinical changes.

Future studies should recruit larger control groups with a more balanced distribution across air mass categories. They should also collect information on air pollution, aeroallergens, and other potential residual confounders, examine whether associations differ by season, and investigate alternative exposure windows, such as five-day meteorological averages.

### 4.4 Conclusion

Synoptic air mass type was associated with distinct peripheral cytokine patterns in schizophrenia, with Tropical air masses corresponding to lower IFN-γ and TNF-α concentrations. The directionally opposite IFN-γ patterns observed in patients and healthy controls may indicate health-status-dependent differences in immune adaptation to environmental conditions. Although no direct association with PANSS severity was detected, these findings support further longitudinal investigation of immunoseasonality as a potential link between meteorological exposure, immune dysregulation, and vulnerability to psychotic exacerbation.

## Data Availability

The raw data supporting the conclusions of this article will be made available by the authors, without undue reservation.
