# Supplementary material for: Air mass advection and immune dysregulation in schizophrenia: an exploratory pilot study
Source: Front Psychiatry. 2026 Oct 19;17:1950134. doi: 10.3389/fpsyt.2026.1950134 (PMC13724432; doi:10.3389/fpsyt.2026.1950134)
Supplement: Supplementary file 1 [file SupplementaryFile1.docx]

**Supplement materials: vector analysis**

| Variable | $V_{NS}$ | $V_{EW}$ | Temperature, °C | Humidity, % | Atmospheric pressure, hPa |
| --- | --- | --- | --- | --- | --- |
| $V_{EW}$ | -.567 (<0.001) | — |  |  |  |
| Temperature, °C | .091 (0.356) | .052(0.598) | — |  |  |
| Humidity, % | **-.458(<0.001)** | **.398 (<0.001)** | **-.302 (0.001)** | — |  |
| Atmospheric Pressure, hPa | .157(0.227) | .109 (0.271) | **-.475 (<0.001)** | **.267 (0.006)** | — |
| Wind stability, *r* | .072(0.942) | -.006(0.947) | **-.310(0.001)** | **-.364(<0.001)** | **.352 (<0.001)** |

Table S1**.** Spearman correlations, N =104. Values represent correlation coefficients ($\rho$) with *p*-values in parentheses.

| Variable | $V_{NS}$, 7-day | $V_{EW}$, 7-day | Temperature, °C | Humidity, % |
| --- | --- | --- | --- | --- |
| $V_{EW}$, 7-day | **-.515 (<0.001)** | — |  |  |
| Temperature, °C | **.408 (<0.001)** | **-.328 (<0.001)** | — |  |
| Humidity, % | **-.626 (<0.001)** | **.26 (0.008)** | **-.281 (0.004)** | — |
| Atmospheric pressure, hPa | **-.304 (0.001)** | **.515 (<0.001)** | **-.707 (<0.001)** | **.177 (0.072)** |

Table S1B. Sensitivity analysis for Table S1. N =104. Values represent correlation coefficients ($\rho$) with *p*-values in parentheses. Seven-day averages showed stronger correlations with meteorological conditions, suggesting that they may provide a more informative proxy of the broader weather environment.

| Cytokine | $V_{NS}$ (North-South) | $V_{EW}$ (East-West) | Wind stability (*r*) |
| --- | --- | --- | --- |
| TNF-α | **.351 (0.001)** | **-.322 (0.003)** | -.011 (0.912) |
| IFN-γ | .142 (0.187) | -.194(0.078) | .179 (0.103) |
| IL-6 | .078 (0.478) | -.088 (0.421) | .063 (0.567) |
| IL-4 | .19 (0.083) | -.069 (0.529) | -.117 (0.289) |

Table S2. Spearman correlations between vector sectors and immune markers (N = 84). Values represent correlation coefficients ($\rho$) are given, with p-values in parentheses. TNF-α correlated with the north and west wind. The anti-inflammatory marker (IL-4) showed no correlation with the winds, which may suggests that the wind selectively promotes pro-inflammatory responses. Cytokines measured in pg/dL.

| Cytokine | $V_{NS}$ (North-South, 7 day mean) | $V_{EW}$ (East-West, 7 day mean) |
| --- | --- | --- |
| TNF-α | **0.346 (0.001)** | **-0.305 (0.005)** |
| IFN-γ | 0.107 (0.333) | **-0.212 (0.053)** |
| IL-6 | 0.122 (0.269) | -0.151 (0.171) |
| IL-4 | .0.113 (0.307) | -0.079 (0.476) |

Table S2B. Sensitivity analysis: Spearman correlations between vector sectors for 7-day means and immune markers (N = 84). Values represent correlation coefficients ($\rho$) are given, with p-values in parentheses. No significant differences were observed between the 3-day and 7-day time windows. Cytokines measured in pg/dL.

Figure S1. Spearman correlation between the north–south wind-vector component (V_NS) and TNF-α concentration. Generated using StataNow 19 SE.

Figure S2. Spearman correlation between the north–south wind-vector component (V_NS) and IFN-γ concentration. Generated using StataNow 19 SE.

Figure S3. Spearman correlation between the north–south wind-vector component (V_NS) and IL-6 concentration. Generated using StataNow 19 SE

Figure S4. Spearman correlation between the north–south wind-vector component (V_NS) and IL-4 concentration. Generated using StataNow 19 SE.

Figure S5. Spearman correlation between the east–west wind-vector component (V_EW) and TNF-α concentration. Generated using StataNow 19 SE.

Figure S6. Spearman correlation between the east–west wind-vector component (V_EW) and IFN-γ concentration. Generated using StataNow 19 SE.

Figure S7. Spearman correlation between the east–west wind-vector component (V_EW) and IL-6 concentration. Generated using StataNow 19 SE.

Figure S8. Spearman correlation between the east–west wind-vector component (V_EW) and IL-4 concentration. Generated using StataNow 19 SE.

| Predictor | PANSS total B; [Cl 95%]; p-value | PANSS positive B; [Cl 95%]; p-value | PANSS negative B; [Cl 95%]; p-value |
| --- | --- | --- | --- |
| Wind $V_{NS}$ | 0.57; [−2.83, 3.96]; 0.739 | −0.67; [−1.97, 0.63]; 0.307 | 0.98; [−0.49, 2.45]; 0.188 |
| Wind $V_{EW}$ | 2.10; [−1.73, 5.92]; 0.278 | −0.11; [−1.57, 1.36]; 0.884 | 1.02; [−0.64, 2.68]; 0.223 |
| CPZ equivalent, mg/day | **0.016; [0.008, 0.025]; <0.001** | **0.006; [0.003, 0.009]; <0.001** | **0.004; [0.0003, 0.008]; 0.034** |
| Smoking, 1 = yes | −1.08; [−9.85, 7.68]; 0.807 | −3.00; [−6.36, 0.36]; 0.079 | **4.09; [0.29, 7.89]; 0.035** |
| BMI, kg/m² | −0.41; [−1.09, 0.27]; 0.236 | −0.03; [−0.30, 0.23]; 0.794 | −0.19; [−0.48, 0.11]; 0.215 |
| Age, years | −0.18; [−0.52, 0.15]; 0.275 | −0.003; [−0.13, 0.12]; 0.958 | −0.03; [−0.18, 0.11]; 0.661 |
| Sex, 1 = men | −5.55; [−14.90, 3.79]; 0.240 | 2.46; [−1.12, 6.04]; 0.175 | **−4.93; [−8.98, −0.88]; 0.018** |
| Constant | 93.39; [68.74, 118.05]; <0.001 | 15.83; [6.39, 25.27]; 0.001 | 25.15; [14.47, 35.84]; <0.001 |
| Model statistics | N = 85; R² = 0.274; Adj. R² = 0.208; F = 4.15; p = .001 | N = 85; R² = 0.239; Adj. R² = 0.170; F = 3.45; p = .003 | N = 85; R² = 0.201; Adj. R² = 0.129; F = 2.77; p = .013 |

Table S3. Weighted linear regression models predicting PANSS scores using local wind-vector components. Values are presented as unstandardized regression coefficients, 95% confidence intervals, and p-values. Models were weighted by the wind direction stability index. Bolded values indicate statistical significance at p < .05. CPZ — chlorpromazine equivalent; PANSS — Positive and Negative Syndrome Scale; BMI — body mass index; CI — confidence interval.

**Supplement materials: air masses models**

Figure S9. Schematic representation of the four air-mass categories used in the study and their predominant directions of advection toward the study region. Arrows indicate generalized source directions and do not represent individual HYSPLIT trajectories.

| Predictor | B [95% CI]; p-value |
| --- | --- |
| Healthy controls vs schizophrenia patients, type 2 | −0.812 [−1.171, −0.452]; 0.001 |
| Type 1 vs type 2 in patients | 0.124 [−0.189, 0.437]; 0.433 |
| Type 3 vs type 2 in patients | 0.038 [−0.181, 0.258]; 0.728 |
| Type 4 vs type 2 in patients | −0.235 [−0.357, −0.112]; <0.001 |
| Healthy controls × type 1 | not estimable |
| Healthy controls × type 3 | −0.221 [−0.648, 0.206]; 0.307 |
| Healthy controls × type 4 | 0.932 [0.452, 1.412]; <0.001 |
| Constant | 3.856 [3.769, 3.943]; <0.001 |
| Model statistics | N = 84; R² = 0.520; Adj. R² = 0.483; F = 38.35; p < .001 |

Reference category: patients with schizophrenia exposed to air mass type 2

Table S4. Interaction model for ln(IFN-γ) according to health status and air mass type
Values are presented as unstandardized regression coefficients, HC3 heteroskedasticity-consistent 95% confidence intervals, and p-values. The outcome variable was natural-log-transformed IFN-γ. The reference category was patients with schizophrenia exposed to air mass type 2. The interaction term for healthy controls × type 1 was not estimable because no healthy controls were observed under type 1 conditions.

| Comparison | Contrast | HC3 SE | t | p-value | 95% CI |
| --- | --- | --- | --- | --- | --- |
| Patients: Polar Maritime Air vs Polar Continental Air | −0.124 | 0.157 | −0.79 | 0.433 | [−0.437, 0.189] |
| Patients: Arctic Air vs Polar Continental Air | −0.086 | 0.182 | −0.47 | 0.639 | [−0.448, 0.276] |
| Patients: Tropical Air vs Polar Continental Air | −0.359 | 0.157 | −2.28 | 0.025 | [−0.672, −0.046] |
| Controls: Polar Maritime Air vs patients: Polar Continental Air | −0.936 | 0.231 | −4.04 | <0.001 | [−1.397, −0.475] |
| Controls: Arctic Air vs patients: Polar Continental Air | −1.118 | 0.161 | −6.93 | <0.001 | [−1.439, −0.797] |
| Controls: Tropical Air vs patients: Polar Continental Air | −0.239 | 0.215 | −1.11 | 0.271 | [−0.668, 0.190] |
| Patients: Arctic Air vs Polar Maritime Air | 0.038 | 0.110 | 0.35 | 0.728 | [−0.181, 0.258] |
| Patients: Tropical Air vs Polar Maritime Air | −0.235 | 0.062 | −3.81 | <0.001 | [−0.357, −0.112] |
| Controls: Polar Maritime Air vs patients: Polar Maritime Air | −0.812 | 0.181 | −4.49 | <0.001 | [−1.171, −0.452] |
| Controls: Arctic Air vs patients: Polar Maritime Air | −0.994 | 0.071 | −13.94 | <0.001 | [−1.136, −0.852] |
| Controls: Tropical Air vs patients: Polar Maritime Air | −0.115 | 0.160 | −0.72 | 0.474 | [−0.433, 0.203] |
| Patients: Tropical Air vs Arctic Air | −0.273 | 0.110 | −2.49 | 0.015 | [−0.492, −0.054] |
| Controls: Polar Maritime Air vs patients: Arctic Air | −0.850 | 0.202 | −4.20 | <0.001 | [−1.253, −0.448] |
| Controls: Arctic Air vs patients: Arctic Air | −1.033 | 0.116 | −8.93 | <0.001 | [−1.263, −0.802] |
| Controls: Tropical Air vs patients: Arctic Air | −0.153 | 0.184 | −0.83 | 0.407 | [−0.519, 0.213] |
| Controls: Polar Maritime Air vs patients: Tropical Air | −0.577 | 0.181 | −3.20 | 0.002 | [−0.936, −0.218] |
| Controls: Arctic Air vs patients: Tropical Air | −0.759 | 0.071 | −10.69 | <0.001 | [−0.901, −0.618] |
| Controls: Tropical Air vs patients: Tropical Air | 0.120 | 0.160 | 0.75 | 0.454 | [−0.198, 0.438] |
| Controls: Arctic Air vs Polar Maritime Air | −0.182 | 0.184 | −0.99 | 0.325 | [−0.549, 0.184] |
| Controls: Tropical Air vs Polar Maritime Air | 0.697 | 0.233 | 2.99 | 0.004 | [0.233, 1.161] |
| Controls: Tropical Air vs Arctic Air | 0.879 | 0.164 | 5.38 | <0.001 | [0.554, 1.205] |

Table S5. Pairwise comparisons of estimated marginal means of ln(IFN-γ).

Pairwise comparisons were derived from the primary OLS interaction model using HC3 heteroskedasticity-consistent standard errors. Comparisons involving healthy controls exposed to Polar Continental Air were not estimable because no controls were observed under this air mass type.

| Air mass type | Schizophrenia patients | Healthy controls | Difference: controls − patients |
| --- | --- | --- | --- |
| Type 1 | 3.980 [3.680, 4.281] | not estimable | — |
| Type 2 | 3.856 [3.769, 3.943] | 3.045 [2.696, 3.393] | −0.812 [−1.171, −0.452]; <0.001 |
| Type 3 | 3.895 [3.694, 4.096] | 2.862 [2.750, 2.974] | −1.033 [−1.263, −0.802]; <0.001 |
| Type 4 | 3.622 [3.535, 3.708] | 3.742 [3.436, 4.047] | 0.120 [−0.198, 0.438]; 0.454 |

Table S6. Estimated marginal means of ln(IFN-γ).
Values are estimated marginal means with HC3 heteroskedasticity-consistent 95% confidence intervals. Between-group differences are presented as healthy controls minus patients with schizophrenia.

| Predictor | B [95% CI]; p-value |
| --- | --- |
| Healthy controls vs schizophrenia patients, type 2 | −0.812 [−1.172, −0.452]; <0.001 |
| Air mass type 3 vs type 2 in patients | 0.038 [−0.181, 0.258]; 0.728 |
| Air mass type 4 vs type 2 in patients | −0.235 [−0.357, −0.112]; <0.001 |
| Healthy controls × type 3 | 0.221 [−0.648, 0.207]; 0.307 |
| Healthy controls × type 4 | 0.932 [0.451, 1.412]; <0.001 |
| Constant | 3.856 [3.769, 3.944]; <0.001 |
| Model statistics | N = 79; R² = 0.521; Adj. R² = 0.488; F(5,73) = 44.48; p < .001 |

Reference category: schizophrenia patients exposed to air mass type 2

Table S7. Sensitivity analysis excluding air mass type 1
Values are presented as unstandardized regression coefficients, HC3 heteroskedasticity-consistent 95% confidence intervals, and p-values. The outcome variable was natural-log-transformed IFN-γ. Air mass type 1 was excluded because no healthy controls were observed under type 1 conditions. The reference category was schizophrenia patients exposed to air mass type 2.

| Predictor | B [95% CI]; p-value |
| --- | --- |
| Healthy controls vs schizophrenia patients, type 2 | −0.772 [−1.151, −0.393]; <0.001 |
| Air mass type 1 vs type 2 in patients | 0.112 [−0.257, 0.481]; 0.547 |
| Air mass type 3 vs type 2 in patients | 0.051 [−0.168, 0.271]; 0.643 |
| Air mass type 4 vs type 2 in patients | −0.229 [−0.344, −0.114]; <0.001 |
| Healthy controls × type 1 | not estimable |
| Healthy controls × type 3 | −0.360 [−0.863, 0.142]; 0.157 |
| Healthy controls × type 4 | 0.854 [0.334, 1.375]; 0.002 |
| Age, years | −0.002 [−0.009, 0.006]; 0.678 |
| Sex, code = 1 | −0.165 [−0.306, −0.025]; 0.022 |
| BMI, kg/m² | −0.002 [−0.012, 0.008]; 0.714 |
| Smoking, 1 = yes | 0.022 [−0.131, 0.175]; 0.775 |
| Constant | 4.063 [3.665, 4.462]; <0.001 |
| Model statistics | N = 84; R² = 0.551; Adj. R² = 0.489; robust F(10,73) = 59.37; p < .001 |

Reference category: schizophrenia patients exposed to air mass type 2

Table S8. Adjusted sensitivity model for ln(IFN-γ).
Values are presented as unstandardized regression coefficients, HC3 heteroskedasticity-consistent 95% confidence intervals, and p-values. The outcome variable was natural-log-transformed IFN-γ. The model was adjusted for age, sex, BMI, and smoking status. The reference category was schizophrenia patients exposed to air mass type 2. The interaction term for healthy controls × type 1 was not estimable because no healthy controls were observed under type 1 conditions.

| Predictor | B [95% CI]; p-value |
| --- | --- |
| Healthy controls vs schizophrenia patients, type 2 | −0.834 [−1.200, −0.469]; <0.001 |
| Air mass type 1 vs type 2 in patients | 0.001 [−0.404, 0.407]; 0.994 |
| Air mass type 3 vs type 2 in patients | −0.027 [−0.296, 0.241]; 0.840 |
| Air mass type 4 vs type 2 in patients | −0.322 [−0.518, −0.125]; 0.002 |
| Healthy controls × type 1 | not estimable |
| Healthy controls × type 3 | −0.282 [−0.787, 0.222]; 0.268 |
| Healthy controls × type 4 | 0.920 [0.378, 1.461]; 0.001 |
| Age, years | −0.002 [−0.009, 0.006]; 0.668 |
| Sex, code = 1 | −0.172 [−0.315, −0.028]; 0.020 |
| BMI, kg/m² | −0.003 [−0.013, 0.007]; 0.548 |
| Smoking, 1 = yes | 0.017 [−0.135, 0.169]; 0.823 |
| 7-day mean temperature, °C | −0.011 [−0.029, 0.006]; 0.201 |
| Constant | 4.229 [3.729, 4.730]; <0.001 |
| Model statistics | N = 84; R² = 0.567; Adj. R² = 0.486; robust F(13,70) = 41.97; p < .001 |

Table S9. Adjusted sensitivity model for ln(IFN-γ) including mean temperature.
Values are presented as unstandardized regression coefficients, HC3 heteroskedasticity-consistent 95% confidence intervals, and p-values. The outcome variable was natural-log-transformed IFN-γ. The model was adjusted for age, sex, BMI, smoking status and mean temperature. The reference category was schizophrenia patients exposed to air mass type 2. The interaction term for healthy controls × type 1 was not estimable because no healthy controls were observed under type 1 conditions

| Predictor | B [95% CI]; p-value |
| --- | --- |
| Healthy controls vs schizophrenia patients, type 2 | −0.806 [−1.192, −0.420]; <0.001 |
| Air mass type 1 vs type 2 in patients | −0.059 [−0.499, 0.382]; 0.791 |
| Air mass type 3 vs type 2 in patients | 0.036 [−0.218, 0.291]; 0.778 |
| Air mass type 4 vs type 2 in patients | −0.340 [−0.564, −0.116]; 0.003 |
| Healthy controls × type 1 | not estimable |
| Healthy controls × type 3 | −0.309 [−0.800, 0.182]; 0.214 |
| Healthy controls × type 4 | 0.893 [0.329, 1.457]; 0.002 |
| Age, years | −0.002 [−0.009, 0.006]; 0.680 |
| Sex, code = 1 | −0.146 [−0.295, 0.002]; 0.053 |
| BMI, kg/m² | −0.002 [−0.012, 0.008]; 0.691 |
| Smoking, 1 = yes | 0.021 [−0.143, 0.184]; 0.801 |
| Spring vs. Winter | −0.168 [−0.416, 0.079]; 0.179 |
| Summer vs. Winter | −0.178 [−0.469, 0.112]; 0.226 |
| Autumn vs. Winter | −0.200 [−0.574, 0.175]; 0.292 |
| Constant | 4.251 [3.777, 4.724]; <0.001 |
| Model statistics | N = 84; R² = 0.561; Adj. R² = 0.494; robust F(11,72) = 56.93; p < .001 |

Table S10. Adjusted sensitivity model for ln(IFN-γ) with seasons.
Values are presented as unstandardized regression coefficients, HC3 heteroskedasticity-consistent 95% confidence intervals, and p-values. The outcome variable was natural-log-transformed IFN-γ. The model was adjusted for age, sex, BMI, smoking status and seasons. The reference category was schizophrenia patients exposed to air mass type 2. The interaction term for healthy controls × type 1 was not estimable because no healthy controls were observed under type 1 conditions

(A)

(B)

Figure S10A, S10B. A supplement for Figure 2 from main text: Points represent back-transformed estimated marginal means, and error bars indicate 95% confidence intervals based on HC3 robust standard errors. Polar Maritime Air - patients with schizophrenia, n = 33, and healthy controls, n = 7; Arctic Air - patients, n = 16, and controls, n = 4; and Tropical Air - patients, n = 11, and controls, n = 8. Polar Continental Air was not displayed because no healthy controls were observed under this condition (patients, n = 5; controls, n = 0). Generated using StataNow 19 SE.
